# Contribution of obesity and cardiometabolic risk factors in developing cardiovascular disease: a population-based cohort study

**DOI:** 10.1038/s41598-022-05536-w

**Published:** 2022-01-28

**Authors:** Mahmood Bakhtiyari, Elham Kazemian, Kourosh Kabir, Farzad Hadaegh, Sepehr Aghajanian, Parham Mardi, Nooshin Taherzadeh Ghahfarokhi, Ali Ghanbari, Mohammad Ali Mansournia, Freidoun Azizi

**Affiliations:** 1 Non-Communicable Diseases Research Center, Alborz University of Medical Sciences, Karaj, Iran; 2 Department of Community Medicine, School of Medicine, Alborz University of Medical Sciences, Karaj, Iran; 3 Prevention of Metabolic Disorders Research Center, Research Institute for Endocrine Sciences, Shahid Beheshti University of Medical Sciences, Tehran, Iran; 4 Student Research Committee, Alborz University of Medical Sciences, Karaj, Iran; 5 Department of Epidemiology and Biostatistics, School of Public Health, Tehran University of Medical Sciences, Pour-Sina Street, Tehran, Iran; 6 Endocrine Research Center, Research Institute for Endocrine Sciences, Shahid Beheshti University of Medical Sciences, Tehran, Iran

**Keywords:** Computational biology and bioinformatics, Cardiology, Diseases, Risk factors

## Abstract

This study aims to assess the effects of central and general adiposity on development of cardiovascular diseases (CVDs) mediated by cardiometabolic risk factors and to analyze their degree of dependency for mediating their effects. To this end, data from the the Tehran Lipid and Glucose Study cohort with 6280 participants were included in this study. The hazard ratios were calculated using a 2-stage regression model in the context of a survival model. Systolic blood pressure (BP), total serum cholesterol, and fasting plasma glucose were designated as mediators. Assessing the interactions revealed that BP was the most important mediator for general ( (HR_NIE_: 1.11, 95% CI 1.17–1.24) and central obesity (CO) (HR_NIE_: 1.11, 95% CI 1.07–1.15) with 60% and 36% proportion of the effects mediated in the total population, respectively. The proportion of mediated risk for all three metabolic risk factors was 46% (95% CI 31–75%) for overweight, 66% (45–100%) for general obesity and 52% (39–87%) for central obesity. BP was the most important mediator for overweight and central obesity in men, comprising 29% and 36% of the risk, respectively. The proportion of the risk mediated through all three metabolic risk factors in women was 23% (95% CI 13–50%) for overweight, 36% (21–64%) for general obesity and 52% (39–87%) for central obesity. Based on the results of this study, cardiometabolic mediators have conciliated more than 60% of the adverse effects of high BMI on CVDs in men. Controlling the metabolic risk factors in women does not efficiently contribute to decreasing CVDs as effectively.

## Introduction

Cardiovascular disease (CVD) is the leading cause of death worldwide^1^. Mortality and morbidity from CVDs are expected to rise in low and middle income countries as well as high income countries over the next few decades^2^. The combination of socio-economic and lifestyle changes has contributed to the development of CVDs over the past decades^3^. Likewise, economic growth, industrialization, increased sedentary lifestyle and nutritional transition has lead to increased prevalence of being overweight and obese. Allied to that, their incidence has doubled and even quadrupled over the last 30 years^4^. It has been shown that overweight and obese individuals have an elevated risk of developing CVDs, particularly those with central obesity^5^. This global increase in prevalence of being overweight and obese and the elevated risk of CVDs has raised concerns in many countries^6^.

The association of obesity with dyslipidemia, hypertension, diabetes, insulin resistance and systemic inflammation which also contribute to risk of developing CVDs themselves, has also been substantiated^5^. However, the mechanisms linking body mass index (BMI) to CVDs have not been clearly understood. The question remains as what proportion of the risks associated with high BMI directly affects cardiovascular disease and how much of it is conciliated by its associated metabolic mediators? In order to clarify this question, we need to first understand how much the effects of obesity could be mediated per se or through other metabolic factors e.g. blood pressure, glucose, cholesterol together or separately. The combined proportion of mediated effect of BP, cholesterol, and diabetes on the association between BMI and incidence of CVDs has been examined in some of the previous studies^7^; However, the effects of individual mediators or possible combination of these risk factors are neglected in these studies^8,9^.

In this study, we have conducted a mediation analysis to examine the degree of effect of overweight and obesity i.e. general and abdominal adiposity on developing CVDs mediated through blood pressure, cholesterol, and blood glucose as single mediators or in varying combinations; along with the assessment of correlation between BMI itself and the mediators. Furthermore, we assessed whether sex-specific analyses could alter overall findings.

## Methods

### Study population

Current research was performed using data derived from The Tehran Lipid and Glucose Study (TLGS) which was a population-based longitudinal cohort study to determine the local epidemiology of non-communicable diseases in Tehran, Iran. In the TLGS, patients were recruited in two phases i.e. the first (1999–2001) and the second (2002–2005) with an approximately 3-year interval^10^.

The eligibility criteria for the current study were as follows: (1) age ≥ 30 years; (2) individuals with at least 1 year follow up; (3) subjects who had data regarding anthropometric and metabolic measurements including BP, waist circumference (WC), fasting plasma glucose (FPG) and 2-h post-challenge plasma glucose (2 h-PCPG), systolic blood pressure (SBP), diastolic blood pressure (DBP) and total cholesterol (TC) at baseline; (4) and data necessary for CVD event assessment during follow-up.

Of the 9560 eligible participants aged ≥ 30 years, those with BMI < 18.5 (n = 113), CVD at baseline (n = 602), history of cancer (n = 57), and hospitalization at baseline (n = 109), and pregnant women (n = 43) and participants lost to follow-up or with missing data regarding metabolic mediators and other covariates (n = 1584) were excluded from the study. Final analyses were performed in 6280 individuals (5357 individuals from exam 1 and 923 new participants from exam 2), who were followed till March 20, 2014. Sequential imputation using chained equations were used to manage missing data in the main variables such as exposures, mediators, and covariates used in the models^11^.

This study obtained ethical approval from the ethics committee at Research Institute for Endocrine Sciences, Shahid Beheshti University of Medical Sciences (IRB approval No.: 240/25) and was conducted in accordance with the Declaration of Helsinki. All participants provided written informed consent.

### Clinical and laboratory measurements

Data related to demographics, smoking habits, physical activity, medical history and medication use were obtained through questionnaires administered by trained physicians^12^. The SBP, DBP and WC were measured at the baseline and every three years intervals which has been described elsewhere^10^. Participants’ fasting venous blood samples were taken after overnight fasting (12–14 h) between 07:00 and 09:00 AM^10^. Serum TC was measured enzymatically via cholesterol oxidase. Measurement protocol for other biochemical variables including FPG and 2hPG, high density lipoprotein cholesterol (HDL-C), and triglyceride (TG) has been described elsewhere^13^.

### Definition of covariates

**Figure 1:**
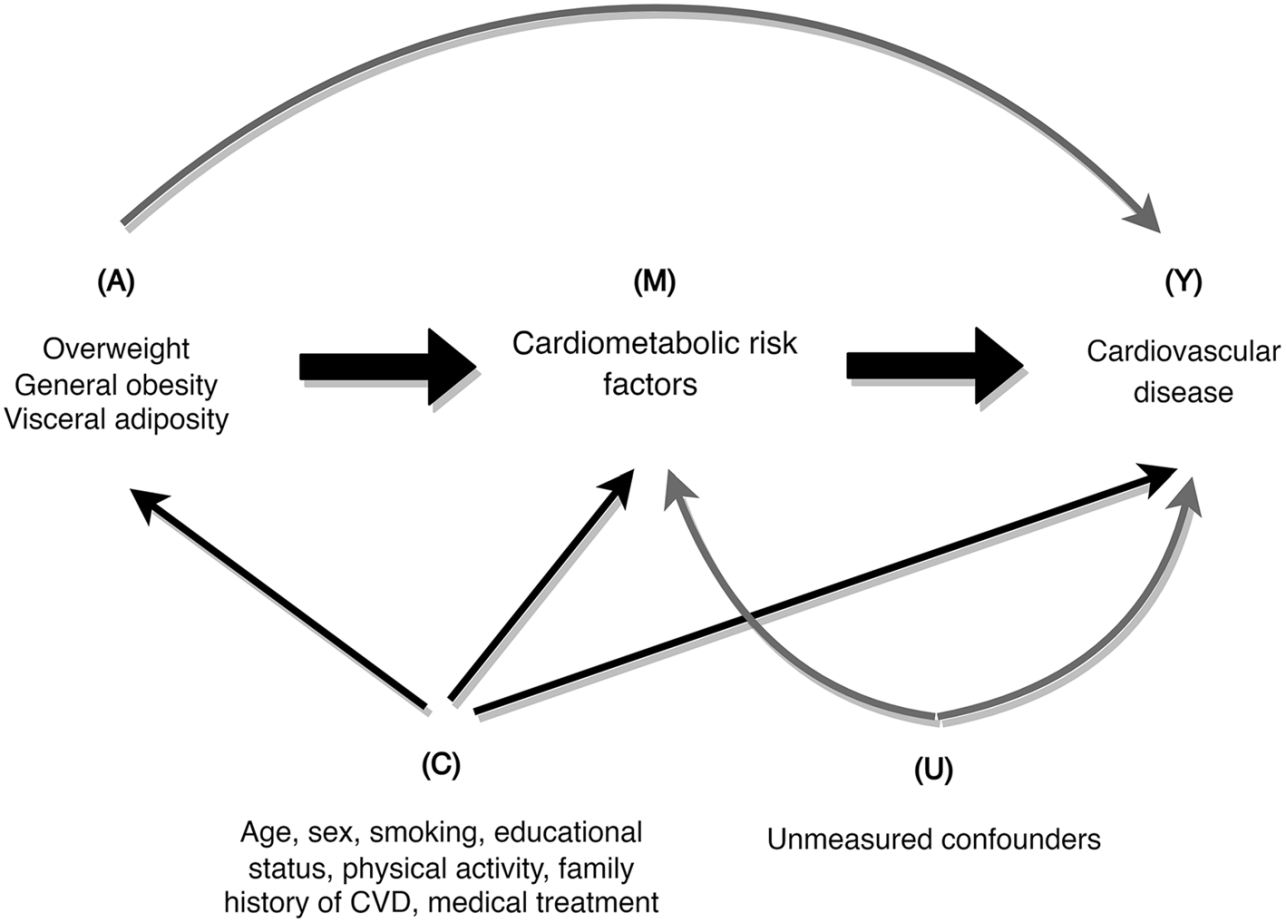
The relationship between exposures (A), mediators (M), outcome (Y), and measured confounders (C) as well unmeasured confounders (U).

Subjects were classified as current smokers if smoked either daily or occasionally, and non-smoker who had never smoked or were ex-smokers. Education levels were categorized as follows: illiterate and those with primary school education, those with and without diploma certificate, and those with a university degree. Central/Visceral adiposity was defined as having WC of ≥ 90 cm for both men and women based on the Iranian National Committee of Obesity reports^14^. Obesity was determined based on BMI and categorized as follows: normal weight (BMI ≤ 25.0 kg/m^2^), overweight (25.0 < BMI < 30.0 kg/m^2^) and general obesity (BMI ≥ 30.0 kg/m^2^). Individuals who had metabolic equivalent task (MET) of less than 600 or exercising less than 3 days a week were considered as insufficiently physically active. Individuals with hypertension were defined as participants with SBP ≥ 140 mmHg, DBP ≥ 90 mmHg, or those receiving antihypertensive medication. Hyperlipidemia was described as total cholesterol ≥ 200 mmol/L. Study variables definition has been detailed elsewhere^12^. In the current study, CVD events were described as a composite count of cases with definite myocardial infarction (MI), probable MI, unstable angina, angiographic-proven coronary heart disease (CHD), CHD death, defnite or possible stroke, transient ischemic attack or cerebrovascular death^15^. The interview guide used in the current investigation was developed for the purpose of this study which has been detailed elsewhere^16^. Demographics, family history of CVDs, and physical activity were regarded as potential factors counfounding the association of central obesity and BMI with CVDs, obesity (both central and general) and mediators, and mediators and CVDs (Fig. 1)^17–23^. Cardiometabolic markers including SBP, TC, and FPG were regarded as mediators^24^.

### Statistical analysis

The differences between baseline continuous and categorical variables in men and women were compared using the *t* test and chi-square test, respectively. In order to specify the relationship between variables and their appropriate scale to be included in the final model, a fractional polynomial model was utilized^25^.

The inverse probability-of-censoring weighting (IPCW) method was employed to adjust for the selection bias resulted from censoring during follow up i.e. loss to follow-up or competing risk^26–28^. We used pooled logistic regression model to assess the inverse probability of loss to follow-up censoring weights in which the censoring variable was regarded as the outcome and other covariates as predictors^29^. The effect of unmeasured mediator-outcomes, confounding direct and indirect effects^30^ as well as the setting of mild and strong confounders were also assessed. Detailed description of the sensitivity analysis is published elsewhere^18^.

A two-stage regression method proposed by VanderWeele was used to estimate the direct and indirect effects^31^. We performed a sensitivity analysis to determine the impact of violations to the no-unmeasured-confounding assumption. The model will provide valid estimates of direct and indirect effects if the occurrence of the outcome is relatively rare, assuming that there are no unmeasured confounding and model misspecifications^30,32^.

First, we fitted three linear regression models, one for each mediator (M), conditional on BMI categories (A) and confounders (C):

1 $${\text{E }}\left[ {{\text{M }}|{\text{ A}},{\text{ C}}} \right] \, = \, \beta_{0} + \, \beta_{{1}} {\text{A}} + \, \beta_{{2}} {\text{C}}$$

We then fitted a Cox proportional hazards regression model for CVD risk on BMI categories (A), mediators (M), a BMI–mediator interaction term, and confounders (C) using age as the time scale:

2 $$\lambda \, \left( {{\text{t }}|{\text{A}},{\text{ M}},{\text{ C}}} \right) \, = \, \lambda_{0} \left( {\text{t}} \right){\text{ exp }}\left( {\theta_{{1}} {\text{A}} + \, \theta_{{2}} {\text{M }} + \theta_{{3}} {\text{AM}} + \, \theta_{{4}} {\text{C}}} \right)$$

where in the equation, λ_0_(t) was the baseline hazard at age t for a normal weight participant when all the mediators and confounders were set to 0 and θ_3_ is the vector of coefficients for the interaction between overweight/obesity and its mediators. The natural direct and indirect effects were estimated using the coefficients of the above regressions. The detailed explanation was provided by Lu et al^24^.

Following convention, the direct and indirect effects at the mean level of confounders in the TLGS cohort was estimated and “proportion of the risk mediated” for each mediator was calculated based on the natural direct and indirect effects, using the formula (HR_TE_–HR_NDE_)/(HR_TE_–1), where HR_TE_ is the total effect hazard ratio and is calculated as HR_TE_ = HR_NDE_ × HR_NIE_^33^. In the final step, a bootstrap with 1000 samples was used for the total, direct, and indirect effects. Statistical analyses were performed using Stata 13.0 MP (Stata corp, College Station, TX, USA) and R 3.04.

## Results

Out of 6280 included subjects, 710 developed CVDs during follow up. The average age of study population was 46.2 ± 11.7 years. The final analysis was performed on 2859 males (45.53%) and 3421 (54.47%) females. No significant differences were found between the men and women in WC, SBP & DBP (*p* > 0.05) while women were more likely to be overweight and have lower education levels as well as higher serum TC compared to men in this study (Table 1). The estimations from the initial parametric regression model showed that being overweight was associated with 61% of the overall increased risk of CVDs. According to our analyses, the indirect hazard ratio for high cholesterol levels was 1.09 (1.06–1.12) with a mediated risk proportion of 22% which was the most important intermediate risk factor in the relation between the overweight and CVDs. Our findings indicated general obesity increased the risk of incident CVDs by 68%. Considering the relation between general obesity and CVDs, the intermediate variables of blood pressure, TC, and FPG were responsible for 17, 14, and 5% of increased risk, respectively; and accounted for the most indirect effects in our analyses. In obese individuals, high blood pressure, TC, and FPG was indirectly accompanied by 33% elevated risk for incident CVDs [HR_NIE_ = 1.33 (1.26–1.42)], comprising 66% of effect of obesity on developing CVDs. Moreover, central obesity increased the risk of CVDs by 59%. The results demonstrated that 52% of total effects of central obesity on developing CVDs were exerted through blood pressure, TC, and FPG mediators (Table 2).

**Table 1:** Baseline characteristics of participants according to gender status, Tehran Lipid and Glucose Study (1999–2015).

Variable	Men (n = 2859)	Women (n = 3421)	Total population (n = 6280)	*P* value*
Age (years)	47.1 ± 12.5	45.5 ± 10.9	46.2 ± 11.7	< 0.001
Body mass index (kg/m^2^)	26.3 ± 3.7	28.6 ± 4.6	27.5 ± 4.4	< 0.001
30 > BMI > 25 (Overweight)	1324 (46%)	1444 (42%)	2768 (44%)	
BMI > 30 (Obese)	455 (16%)	1208 (35%)	1663 (26%)	
Waist circumference(cm)	90.9 ± 10.3	90.4 ± 11.9	90.6 ± 11.2	0.095
WC ≥ 90 (Visceral adiposity)	1583 (55%)	1793 (52%)	3376 (54%)	0.019
Systolic blood pressure (mmHg)	120.9 ± 18.4	120.1 ± 19.3	120.5 ± 18.9	0.08
Diastolic blood pressure (mmHg)	78.5 ± 11.2	78.8 ± 10.6	78.6 ± 10.9	0.43
Hypertension (yes)	634 (22%)	844 (25%)	1478 (24%)	0.02
Fasting plasma glucose (mg/dl)	5.29 ± 1.17	5.21 ± 1.1	5.25 ± 1.13	0.009
Diabetes mellitus (yes)	196 (7%)	256 (7%)	452 (7%)	0.33
Triglycerides (mmol/L)	2.19 ± 1.56	1.91 ± 1.18	2.04 ± 1.37	< 0.001
Total cholesterol (mmol/L)	5.38 ± 1.1	5.63 ± 1.2	5.51 ± 1.16	< 0.001
Total cholesterol ≥ 200 mmol/L (Hyperlipidemia)	46 (1%)	115 (3%)	161 (3%)	< 0.001
HDL-cholesterol (mmol/L)	0.98 ± 0.24	1.15 ± 0.28	1.07 ± 0.27	< 0.001
Family history of CVDS (%)	417 (14.59)	606 (17.71)	1023(16.29)	0.001
Low physical activity (%)	2061 (72.09)	2402 (70.21)	4463(71.07)	0.10
**Education (%)**				
Illiterate/primary school	824 (28.82)	1563 (45.6)	2387(38.01)	< 0.001
Below diploma/diploma	1497 (52.36)	1595 (46.62)	3092(49.24)	
Higher than diploma	538 (18.82)	263 (7.69)	801(12.75)	
**Smoking (%)**				
Never	1528 (53.45)	3196 (93.42)	4724(75.22)	< 0.001
Past	453 (15.84)	68 (1.99)	521(8.3)	
Current	878 (30.71)	157 (4.59)	1035(16.48)	

*Differences in continuous and categorical variables between males and females were assessed using the independent *t*-test and Chi-square test, respectively.

**Table 2:** Total, direct, and indirect effects of overweight and adiposity on cardiovascular diseases (CVDs) using a parametric method without considering exposure-mediator interaction.

Exposures	Mediators	Total effect^a,b^	Natural direct effect	Natural indirect effect	Proportion mediated^c^ (95% CI)
		HR (95% CI)	HR (95% CI)	HR (95% CI)	
Overweight	Blood pressure (mmHg)	1.61 (1.34–1.95)	1.42 (1.18–1.73)	1.08 (1.06–1.11)	21 (14–37)
	Cholesterol (mmol/L)		1.5 (1.23–1.81)	1.09 (1.06–1.12)	22 (15–34)
	Glucose (mmol/L)		1.53 (1.27–1.86)	1.02 (1.03–1.04)	8 (5–15)
	Blood pressure, cholesterol, and glucose		1.29 (1.07–1.58)	1.19 (1.15–1.24)	46 (31–75)
General obesity	Blood pressure (mmHg)	1.68 (1.35–2.08)	1.37 (1.08–1.70)	1.17 (1.11–1.21)	38 (26–70)
	Cholesterol (mmol/L)		1.5 (1.21–1.85)	1.14 (1.10–1.18)	29 (20–45)
	Glucose (mmol/L)		1.58 (1.26–1.96)	1.05 (1.03–1.07)	12 (7–20)
	Blood pressure, cholesterol, and glucose		1.20 (0.96–1.52)	1.33 (1.26–1.42)	66 (45–100)
Visceral adiposity	Blood pressure (mmHg)	1.59 (1.33–1.85)	1.40 (1.18–1.63)	1.10 (1.07–1.13)	27 (18–43)
	Cholesterol (mmol/L)		1.44 (1.19–1.67)	1.09 (1.06–1.12)	23 (16–38)
	Glucose (mmol/L)		1.50 (1.25–1.74)	1.04 (1.02–1.06)	11 (8–19)
	Blood pressure, cholesterol, and glucose		1.25 (1.03–1.46)	1.22 (1.17–1.27)	52 (39–87)

*MI* body mass index; *CI* confidence interval; *HR* hazard ratio; *WC* waist circumference.

^a^Compared with normal-weight participants for general adiposity and WC < 90 cm as a reference for central adiposity.

^b^All models were adjusted for age, gender, smoking, physical activity level, educational status, and family history of CVDs.

^c^The direct, indirect, and total effects were estimated for each bootstrap resampling.

Median duration of follow up was 13.9 years in the included participants. The proportion of censored cases during the follow-up period was 8.36%. The result of IPCW demonstrated that the amount of participants censored due to loss to follow-up for increased BMI, adiposity, and central adiposity was less than 5% (Supplementary Table S1). Furthermore, multiple imputation of missing data didn't show any significant differences (less than 5%) with observed estimates (see Supplementary Table S2).

Tables 3 and 4 demonstrate the results of the gender-based parametric model. The general estimated effects of the overwight, general and visceral obesity were associated with 11, 28 and 17% decrease in risk of developing CVDs in men compared to the whole population. In addition, our finding showed high blood pressure, TC and FPG with an intermediate contribution percentage of 29, 24 and 11%, respectively, were the most important cardio-metabolic intermediate variables in the relation between the overweight and CVDs in men. We also observed that 60% of the association of being overweight with incident CVDs was mediated by the concurrent presence of hypertension, high TC, and FPG. It is worth noting that the same effect for the whole studied population was calculated to be around 46% (Tables 3, 4).

**Table 3:** Total, direct, and indirect effects of overweight and adiposity on cardiovascular diseases (CVDs) using a parametric method not considering exposure-mediator interaction in men.

Exposures	Mediators	Total effect^a,b^	Natural direct effect	Natural indirect effect	Proportion mediated^c^ (95% CI)
		HR (95% CI)	HR (95% CI)	HR (95% CI)	
Overweight	Blood pressure (mmHg)	1.50 (1.22–1.86)	1.32 (1.07–1.64)	1.10 (1.06–1.14)	29 (16–62)
	Cholesterol (mmol/L)		1.40 (1.13–1.75)	1.09 (1.05–1.13)	24 (14–48)
	Glucose (mmol/L)		1.42 (1.15–1.77)	1.04 (1.02–1.05)	11 (6–26)
	Blood pressure, cholesterol, and glucose		1.17 (0.93–1.47)	1.23 (1.17–1.29)	60 (30–100)
General obesity	Blood pressure (mmHg)	1.40 (1.06–1.85)	1.12 (0.82–1.52)	1.11 (1.17–1.24)	60 (1–100)
	Cholesterol (mmol/L)		1.27 (0.95–1.67)	1.14 (1.08–1.22)	40 (22–99)
	Glucose (mmol/L)		1.28 (0.97–1.70)	1.06 (1.03–1.10)	22 (10–99)
	Blood pressure, cholesterol, and glucose		0.95 (0.70–1.28)	1.39 (1.29–1.51)	98 (10–100)
Visceral adiposity	Blood pressure (mmHg)	1.42 (1.16–1.73)	1.24 (1.02–1.56)	1.11(1.07–1.15)	36 (19–83)
	Cholesterol (mmol/L)		1.30 (1.05–1.60)	1.09 (1.06–1.13)	29 (15–67)
	Glucose (mmol/L)		1.34 (1.11–1.66)	1.04 (1.02–1.06)	14 (7–30)
	Blood pressure, cholesterol, and glucose		1.11 (0.90–1.41)	1.25 (1.18–1.32)	71 (41–100)

*MI* body mass index; *CI* confidence interval; *HR* hazard ratio; *WC* waist circumference.

^a^Compared with normal-weight participants for general adiposity and WC < 90 cm as a reference for central adiposity.

^b^All models were adjusted for age, gender, smoking, physical activity level, educational status, and family history of CVDs.

^c^The direct, indirect, and total effects were estimated for each bootstrap resampling.

**Table 4:** Total, direct, and indirect effects of overweight and adiposity on cardiovascular diseases (CVDs) using a parametric method not considering exposure-mediator interaction in women.

Exposures	Mediators	Total effect^a,b^	Natural direct effect	Natural indirect effect	Proportion mediated (95% CI)
		HR (95% CI)	HR (95% CI)	HR (95% CI)	
Overweight	Blood pressure (mmHg)	1.91 (1.32–2.93)	1.76 (1.21–2.75)	1.05 (1.02–1.08)	10 (4–22)
	Cholesterol (mmol/L)		1.80 (1.26–2.81)	1.06 (1.03–1.10)	12 (1–25)
	Glucose (mmol/L)		1.83 (1.25–2.79)	1.02 (1.01–1.03)	5 (2–11)
	Blood pressure, cholesterol, and glucose		1.64 (1.13–2.56)	1.11 (1.07–1.17)	23 (13–50)
General obesity	Blood pressure (mmHg)	2.13 (1.44–3.20)	1.82 (1.23–2.79)	1.13 (1.07–1.19)	22 (11–41)
	Cholesterol (mmol/L)		1.96 (1.35–3.03)	1.08 (1.04–1.13)	14 (7–26)
	Glucose (mmol/L)		2.04 (1.39–3.04)	1.03 (1.01–1.03)	6 (3–14)
	Blood pressure, cholesterol, and glucose		1.70 (1.15–2.64)	1.21 (1.14–1.31)	36 (21–64)
Visceral adiposity	Blood pressure (mmHg)	1.72 (1.39–2.39)	1.59 (1.16–2.20)	1.08 (1.04–1.12)	18 (10–40)
	Cholesterol (mmol/L)		1.62 (1.19–2.27)	1.05 (1.02–1.08)	13 (5–25)
	Glucose (mmol/L)		1.64 (1.19–2.24)	1.03 (1.01–1.06)	9 (4–22)
	Blood pressure, cholesterol, and glucose		1.47 (1.07–2.06)	1.14 (1.09–1.21)	31 (18–69)

*MI* body mass index; *CI* confidence interval; *HR* hazard ratio; *WC* waist circumference.

^a^Compared with normal-weight participants for general adiposity and WC < 90 cm as a reference for central adiposity.

^b^All models were adjusted for age, gender, smoking, physical activity level, educational status, and family history of CVDs.

^c^The direct, indirect, and total effects were estimated for each bootstrap resampling.

The results of the association between obesity and CVDs revealed that the estimated total effects were lower in men compared to women. The presence of cardiometabolic risk factors in men was associated with less harmful effects on the incidence of CVDs compared to women. We found that the effects of obesity on incident CVDs in men having the three analyzed cardiometabolic risk factors were not mediated via direct natural effects but mostly through causal route (intermediate share 98%).

Furthermore, our analyses indicate the most crucial intermediate variables in men with abdominal obesity were the same as those in overweight and obese individuals and was 71%. We observed that controlling intermediate risk factors in men with general obesity came with better results in controlling and preventing CVDs rather than those with abdominal obesity. The results of the parametric model showed that overweight, general, and abdominal obesity were associated with higher risks of developing CVDs in women compared to men and exerted their effects mostly independent of the cardiometabolic risk factors. The increased risk was 40% higher for overweight, 60% for general obesity and 30% for abdominal obesity compared to men. The direct natural effects had a more significant proportion of the whole effects and the mediators played a weaker role in conducting the harmful effects on developing CVDs in women. For example, in the obese individuals, the intermediate contribution of high BP, TC, and FPG in incident CVDs, if co-occurred, was 36% among women compared to 98% in men.

Table 5 illustrates the parametric model analyses indicating the interaction between the exposures, namely, overweight, general and visceral obesity and the intermediate variables were not statistically significant. The reason behind the minute changes of the whole effects in this table compared to the results observed in Table 1 (Table 2) was the incorporation of possible interactions in models. There were no interactions between any mediator and the exposure in the models. The sensitivity analysis for unmeasured mediator-outcome confounding suggested the variations were less than 5% in the two scenarios (Supplementary Table S3).

**Table 5:** Total, direct, and indirect effects of overweight and adiposity on cardiovascular diseases (CVDs) using a parametric method considering exposure-mediator interaction.

Exposures	Mediators	Total effect^a,b^	Natural direct effect	Natural indirect effect	Multiplicative interaction (95% CI)
		HR (95% CI)	HR (95% CI)	HR (95% CI)	
Overweight	Blood pressure (mmHg)	1.50 (1.29–1.90)	1.46 (1.17–1.74)	1.08 (1.05–1.12)	1 (0.99–1.07)
	Cholesterol (mmol/L)	1.64 (1.35–1.94)	1.51 (1.24–1.82)	1.08 (1.04- 1.12)	0.93 (0.83–1.04)
	Glucose (mmol/L)	1.57 (1.30–1.87)	1.53 (1.27–1.82)	1.03 (1.01–1.04)	0.95 (0.87–1.02)
	Blood pressure, cholesterol, and glucose	1.61 (1.30–1.94)	1.38 (1.10–1.67)	1.17 (1.12–1.22)	1 (0.99–1.007)
					0.90 (0.80–1.10)
					0.92 (0.85–1.01)
General obesity	Blood pressure (mmHg)	1.69 (1.36–2.07)	1.50 (1.20–1.91)	1.12 (1.05–1.19)	1 (0.99–1.00)
	Cholesterol (mmol/L)	1.67 (1.36–2.05)	1.42 (1.15–1.78)	1.17 (1.11–1.24)	0.93 (0.83–1.04)
	Glucose (mmol/L)	1.60 (1.31–1.94)	1.48 (1.21–1.79)	1.07 (1.04–1.11)	0.95 (0.87–1.02)
	Blood pressure, cholesterol, and glucose	1.66 (1.33–2.07)	1.20 (0.94–1.54)	1.37 (1.26–1.50)	1.08 (0.99–1.07)
					0.90 (0.80–1.01)
					1.02 (0.85–1.04)
Visceral adiposity	Blood pressure (mmHg)	1.59 (1.34–1.79)	1.41 (1.18–1.65)	1.07 (1.07–1.13)	1.01 (0.98–1.04)
	Cholesterol (mmol/L)	1.57 (1.31–1.82)	1.42 (1.17–1.67)	1.09 (1.06–1.15)	1.02 (0.97–1.08)
	Glucose (mmol/L)	1.60 (1.29–1.84)	1.51 (1.26–1.76)	1.05 (1.04–1.07)	1.00 (0.99–1.01)
	Blood pressure, cholesterol, and glucose	1.63 (1.35–1.83)	1.22 (1.03–1.42)	1.23 (1.16–1.23)	0.98 (0.99–1.002)
					0.99 (0.96–1.03)
					1.00 (0.99–1.01)

*MI* body mass index; *CI* confidence interval; *HR* hazard ratio; *WC* waist circumference.

^a^Compared with normal-weight participants for general adiposity and WC < 90 cm as a reference for central adiposity.

^b^All models were adjusted for age, gender, smoking, physical activity level, educational status, and family history of CVDs.

^c^The direct, indirect, and total effects were estimated for each bootstrap resampling.

A separate analysis was done while taking into account the effect of medications for diabetes mellitus, hypertension, and dyslipidemia (hypercholesterolemia) to provide proportion of mediated effects adjusted for medications for each mediator (Supplementary Table S4).

## Discussion

The results of this study demonstrated that a proportion of increased risk of CVDs in overweight individuals and subjects with increased general or visceral adiposity is exerted independent of the analyzed intermediate metabolic risk factors, which was greater in women compared to men. Cardiometabolic risk factors, including hypertension, high FPG, and TC levels contributed to 46, 66, and 52% of the increased risk of incidence of CVDs in overweight and obese individuals, respectively. The most important variable intermediating the relation between overweight and CVDs was high serum TC concentration (22%), while hypertension was identified as the most important factor which mediated the effects of general obesity on CVD incidence in our data (38%). Hypertension was also the most important mediator variable between visceral obesity and development of CVDs. In men, a total of 60% of the incresaed risk of CVDs of overweight subjects, 98% of those with general obesity, and 71% of those visceral obesity were mediated via hypertension and high serum TC and FPG. However, in women, the effects of obesity on incidence of CVDs were mostly exerted directly and independent of the risk factors. An interesting observation in this study was the effect of therapeutic medications in proportion of mediated risk for each risk factor. The secondary analysis (Supplementary Table S4) in which the results were adjusted for the use of medications for hypertension, diabetes mellitus, and hypercholestrolemia, led to different proportion of mediated risk for mediators.

While BMI is considered as an excellent index of general obesity, using WC to assess central obesity is regarded as a better index to show visceral fat deposition and consequently worsening metabolic profile^34^. Several studies have been conducted on the effects of central obesity on cardiovascular events^35,36^. In a study by Bogers et al. showed overweight can increase the effects of high cholesterol and hypertension on CVDs up to 45%^37^. It was also reported that the overweight and obesity increased the mortality risk caused by coronary heart diseases (CHDs) in both patients previously diagnosed with CHDs and those without a history of CHDs; although this effect is faster in the latter group^38^. In a study by Jousilahti et al. showed that each 1 kg increase in weight was accompanied by 1–1.5% elevated risk of mortality caused by CHDs^39^. In another study performed in 221,934 people in 17 countries indicated BMI, WC, and waist to hip ratio, either alone or in combination with other mediators, did not increase the risk of CVDs after further adjustment for baseline SBP, history of diabetes, total and high-density lipoprotein (HDL) cholesterol levels^40^.

In the study conducted by Kazempour‐Ardebili on Tehran residents aged ≥ 65 years revealed that visceral rather than general obesity contributes to development of CVDs and CHDs which was partially mediated via cardiometabolic risk factors, specifically hypertension^41^. However, the small sample size and lack of use of standardized models of causal mediation analyses could have contributed to the varying results, considering that both studies were performed in the the same population.

The incidence and prevalence of general and visceral obesity has risen in the Iranian adults in the recent years^42^ and the current interventions and policies have failed to control this health problem.; Therefore, there is a growing interest to recognize the causal patterns of obesity and its its metabolic mediators^43^. Investigaring the causal patterns in which how and from what route the potential risk factors exert their protective or harmful effects on the outcome is called mediation analysis^44^. The primary purpose of these methods is delineate the interventions’ effects with removing components that do not have any impact on the outcome^45^.

In a study by Lu et al. revealed that hypertension mediated 22% and 36% of adverse effects of overweight and abdominal obesity on CHDs, respectively; while for obesity, an elevated blood sugar levels, accounted for 65% of its effects on the incident outcome as the most important mediator. Furthermore, it was shown that overweight, obesity, and a waist circumference > 90 cm contributed their adverse effects on CHDs through the three cardiometabolic risk factors including hypertension (54%), elevated blood sugar (81%), and cholesterol (62%)^24^.

Lu et al. in a pooled analyses of 97 prospective cohort studies reported that each 5 kg/m^2^ higher BMI was accompanied with 27% elevated risk for CHDs which was mediated through high blood pressure, cholesterol, and glucose. In addition, it is indicated that obesity and overweight increase the risk for CHDs independent of these selected metabolic risk factors by 54%^17^.

Several studies have been performed to investigate the the impacts of visceral obesity on incident CVDs^2,3^. For example in a study by Bakhtiyari et al. the relationship between obesity and CVDs was determined by employing nonparametric methods. It was revealed that the essential mediators which linked the relationship between overweight, general, and visceral obesity with CVDs, were hypertension (PM = 22), high cholesterol (PM = 65) and blood glucose concentations (PM = 36). They also showed that 81% of the effects caused by obesity on incident CVDs were conducted via three mediators including hypertension, high cholesterol, and blood glucose levels. The nonparametric methods used in the study by Bakhtiyari et al. may contribute to the different results of their analyses^18^.

The findings of current study suggested that the general and central obesity indices were accompanied by the increased risk of CVDs, independent of the previously mentioned cardiometabolic risk factors. While our findings contradict the results of the previous studies^41,46^, It could be postulated that failure to adjust for potential confounding variables and different age groups in prior studies may have contributed to the observed dissimilarities^47^. Another factor that may have influenced the results of sties is the age pattern of the study population. It is of note to say that BMI is not a good index for adiposity in the elderly, as skeletal muscle mass reduce and abdominal obesity increases with aging^48^.

Different mechanisms link general and abdominal obesity to CVD via cardiometabolic risk factors. When excessive fat accumulates, even in the absence of systematic hypertension and underlying cardiac disease, remarkable changes in the structure and function of the heart occurs. To overcome the metabolic needs, circulating blood and plasma volume, as well as cardiac output increases. The increase in blood volume leads to an increase in the venous return to the left ventricle, which will lead to cardiac chambers diastolic compliance reduction and an increase in the left ventricle filling time and left ventricle enlargement. As long as left ventricular hypertrophy is synced with the left ventricular hypertrophy(LVH), the systolic activity of the heart is preserved. When LVH cannot keep up with the progressive increase in heart size, the increased cardiac wall pressure may lead to systolic dysfunction. An increase in systematic and pulmonary blood pressure (left ventricle failure and chronic hypoxia) and CHDs can all occur due to the impact of obesity on the structure and function of the heart. Also, the risk of sudden cardiac death increases with progression of obesity ^49^. Another mechanism is the release of bioactive mediators from the adipose tissue that, by acting on blood lipids, blood pressure, inflammation, and coagulation, will eventually lead to blood vessel dysfunction and atherosclerosis^50^.

After sensitivity analysis and considering (U) energy-adjusted glycemic load as an unmeasured confounder variable in this study, the results did not show a tangible change, and this is a support to the validity of the results observed in the current analyses. No difference was indicated in the sensitivity analysis conducted Lu et al.^17^.

The strength of the methods used in the current study are freedom in input exposure and outcome types, intermediate variable count, applicability in most basic regression equations including survival models, and the ability to assess any interaction between exposure and the mediators.

This study however had initially met several limitations, some of which were addressed throughout the text. The wide intervals for the proportion of mediated risks estimated indices calculated in this study may be attributable to high variation and instability of the indices themselves^51^. Furthermore, the small sample size^24^ and the changes in the levels of the measured risk factors at baseline and during follow up may have confound our findings. Moreover, information bias due to incomplete data recording and assumption of “no correlation” between the potential risk factors for CVDs should also be considered as limiting factors in the current study. For example, studies have reported that an increase in patients’ blood glucose levels could lead to an increase in their blood pressure which may subsequently affect kidneys function and circulating cholesterol levels^52^. In order to achieve a valid estimation of the direct and indirect natural effects in the current study, it was assumed that there is no confounding variable between the following relations; (A): BMI (or WC) and CVDs, (B): Mediator and CVDs, (C): BMI (or WC) and mediator, and that (D): No confounder is present in the relation between the mediators and CVDs that is being affected by the exposures. Individuals with a BMI < 18.5 and history of hospital admission were excluded to avoid any confounding effects between BMI (or WC) and CVDs. The effects of unmeasured mediator–CVD confounding was determined by calculating a bias factor which revealed no significant changes in the estimations were. To assess the assumption “*C*,” the main reasons relating to BMI and mediators such as physical activity, smoking, education, and history of CVDs were included in the analyses. Finally, assumption “*D*” was not verified if, for instance, physical activity affects both hypertension (diabetes) and CVDs wherase it was affected by obesity. To address this, in one instance, the physical activity was treated as as a new variable, whereas in another instance, its direct effects were estimated using marginal structural and structural nested models which do not divide the effects into direct and indirect ones.

## Conclusions

In conclusion, the results of this study demonstrated that the negative effects of obesity in development of CVDs were mostly via hyprtension, high blood glucose, and cholesterol levels in men. Thus management of these three cardiometabolic factors should be considered as alternative, effective interventions compared to normally inefficient modalities targeting obesity in reduction of CVDs in men^53–55^. However, the greater role of direct effects of obesity in women should not be neglected. In other words, Obesity is considered as a crucial risk factor for development of CVDs. Current strategies for controlling weight including behavioral, medication and surgical interventions have been subject of criticism due to their insufficient questionable efficacy^56^. On the other hand, hypertention and dyslipidemia could be effectively managed via antihypertensive or lipid-lowering agents. Therefore, understanding the casual pathway of obesity and its cardiometabolic risk factors could provide effective and practical solutions to decrease the obesity related comorbidities^57–59^.

## Supplementary Information

Supplementary Table S1.

Supplementary Table S2.

Supplementary Table S3.

Supplementary Table S4.
