# Supplementary material for: Contribution of obesity and cardiometabolic risk factors in developing cardiovascular disease: a population-based cohort study
Source: Sci Rep. 2022 Jan 28;12:1544. doi: 10.1038/s41598-022-05536-w (PMC8799723; doi:10.1038/s41598-022-05536-w)
Supplement: Supplementary file 1 — Supplementary Table S1. [file 41598_2022_5536_MOESM1_ESM.docx]

Supplementary Table 1. Total, direct, and indirect effects of general and central adiposity^1^ on CVDs adjusted for censoring weight, using inverse probability weighting-of-censoring method (IPCW), Tehran Lipid and Glucose Study (n=6280)

| **Exposures** | **Mediators** | **Total effect^2^** | **Natural direct effect** | **Natural indirect effect** |
| --- | --- | --- | --- | --- |
|  |  | **HR (95% CI)** | **HR (95% CI)** | **HR (95% CI)** |
| Overweight | Blood pressure (per 10 mmHg) | 1.61  (1.34-2.01) | 1.42(1.16-1.81) | 1.09(1.06-1.15) |
|  | Cholesterol (per 1 mmol/l) |  | 1.49(1.22-1.82) | 1.08(1.06-1.14) |
|  | Glucose (per 1 mmol/l) |  | 1.53(1.23-1.88) | 1.02(1.01-1.06) |
|  | Blood pressure, cholesterol, glucose |  | 1.28 (1.08-1.57) | 1.19(1.13-1.24) |
| General obesity | Blood pressure (per 10 mmHg) | 1.67  (1.36-2.10) | 1.37(1.08-1.70) | 1.17(1.11-1.22) |
|  | Cholesterol (per 1 mmol/l) |  | 1.50(1.21-1.86) | 1.15(1.10-1.18) |
|  | Glucose (per 1 mmol/l) |  | 1.57(1.26-1.95) | 1.05(1.03-1.07) |
|  | Blood pressure, cholesterol, glucose |  | 1.20(0.95-1.53) | 1.33(1.26-1.43) |
| Visceral adiposity | Blood pressure (per 10 mmHg) | 1.59  (1.33-1.89) | 1.40(1.17-1.64) | 1.10(1.07-1.13) |
|  | Cholesterol (per 1 mmol/l) |  | 1.45(1.18-1.67) | 1.09(1.06-1.12) |
|  | Glucose (per 1 mmol/l) |  | 1.51(1.25-1.73) | 1.04(1.02-1.06) |
|  | Blood pressure, cholesterol, glucose |  | 1.25(1.03-1.45) | 1.21(1.18-1.28) |

1. Compared with normal-weight participants for general adiposity and WC<90cm as a reference for central adiposity
2. All models were adjusted for age, gender, smoking, physical activity level, educational status, and family history of CVDS.
3. The direct, indirect, and total effects were estimated for each bootstrap resample.
