# Supplementary material for: Contribution of obesity and cardiometabolic risk factors in developing cardiovascular disease: a population-based cohort study
Source: Sci Rep. 2022 Jan 28;12:1544. doi: 10.1038/s41598-022-05536-w (PMC8799723; doi:10.1038/s41598-022-05536-w)
Supplement: Supplementary file 2 — Supplementary Table S2. [file 41598_2022_5536_MOESM2_ESM.docx]

Supplementary Table 2. Total, Direct, and Indirect Effects of General and Central adiposity^1^ on CVD for Metabolic Mediators after the multiple imputation of missing data without considering exposure-mediator interaction, Tehran Lipid and Glucose Study

| **Exposures** | **Mediators** | **Total effect^2^** | **Natural direct effect** | **Natural indirect effect** | **PM^3^**  **(95% CI)** |
| --- | --- | --- | --- | --- | --- |
|  |  | **HR (95% CI)** | **HR (95% CI)** | **HR (95% CI)** |  |
| **Overweight** | Blood pressure (per 10 mmHg) | 1.49  (1.16-1.76) | 1.36(1.08-1.52) | 1.09 (1.03-1.17) | 26% (10%-46%) |
|  | Cholesterol (per 1 mmol/l) |  | 1.38 (1.07-1.66) | 1.09(1.02-1.14) | 23% (11%-51%) |
|  | Glucose (per 1 mmol/l) |  | 1.40 (1.10-1.70) | 1.06 (1.01-1.11) | 16% (8%-33%) |
|  | Blood pressure, cholesterol, glucose |  | 1.21 (0.95-1.56) | 1.21 (1.16-1.31) | 53% (33%-83%) |
| **General obesity** | Blood pressure (per 10 mmHg) | 1.58  (1.20-1.79) | 1.48 (1.12-2.02) | 1.08 (0.89-1.27) | 20% (5%-60%) |
|  | Cholesterol (per 1 mmol/l) |  | 1.38 (1.04-1.84) | 1.16 (0.96-1.35) | 35% (13%-74%) |
|  | Glucose (per 1 mmol/l) |  | 1.43 (1.1-1.81) | 1.12 (0.96-1.27) | 28% (12%-62%) |
|  | Blood pressure, cholesterol, glucose |  | 1.23 (0.88-1.65) | 1.30 (1.07-1.58) | 61% (25%-92%) |
| **Visceral adiposity** | Blood pressure (per 10 mmHg) | 1.52  (1.20-1.72) | 1.33 (1.10-1.64) | 1.14 (1.07-1.20) | 37% (22%-63%) |
|  | Cholesterol (per 1 mmol/l) |  | 1.41 (1.16-1.70) | 1.08 (1.03-1.12) | 21% (13%-42%) |
|  | Glucose (per 1 mmol/l) |  | 1.36 (1.13-1.76) | 1.11(1.06-1.15) | 29% (19%-52%) |
|  | Blood pressure, cholesterol, glucose |  | 1.16 (0.97-1.45) | 1.31 (1.21-1.40) | 67% (48%-96%) |

1. Compared with normal weight participants for general adiposity and WC<90cm as referent for central adiposity
2. All models were adjusted for age, gender, smoking, physical activity level, educational status and family history of CVD
3. Proportion mediated
