# Supplementary material for: Contribution of obesity and cardiometabolic risk factors in developing cardiovascular disease: a population-based cohort study
Source: Sci Rep. 2022 Jan 28;12:1544. doi: 10.1038/s41598-022-05536-w (PMC8799723; doi:10.1038/s41598-022-05536-w)
Supplement: Supplementary file 3 — Supplementary Table S3. [file 41598_2022_5536_MOESM3_ESM.docx]

Supplementary Table 3. Sensitivity analyses of the impact of unmeasured mediator-CVD confounding (U) on hazard ratios (HR) for direct, indirect effects, and proportion mediated (PM) of overweight, general obesity and central obesity on CVD for three metabolic mediators combined

| **Exposures** | **Observed analysis**  (No unmeasured confounding, HR_U_=1)^2^ | | | **Mild unmeasured confounding**  (HR_U_=1.1) | | | **Strong unmeasured confounding**  (HR_U_=1.98) | | |
| --- | --- | --- | --- | --- | --- | --- | --- | --- | --- |
|  | HR_NDE_ | HR_NIE_ | PM (%) | HR_NDE_ | HR_NIE_ | PM (%) | HR_NDE_ | HR_NIE_ | PM (%) |
| **Overweight** | 1.29 (1.07-1.58) | 1.19 (1.15-1.24) | 46 (31-75) | 1.30 (1.07-1.59) | 1.19(1.16-1.25) | 45 (31-74) | 1.24 (1.03-1.52) | 1.15 (1.11-1.19) | 44 (32-71) |
| **General obesity** | 1.20 (0.96-1.52) | 1.33 (1.26-1.42) | 66 (45-100) | 1.21 (0.96-1.53) | 1.34 (1.27-1.43) | 66 (44-99) | 1.16 (0.92-1.46) | 1.28 (1.21-1.36) | 69 (46-100) |
| **Visceral adiposity** | 1.25 (1.03-1.46) | 1.22 (1.17-1.27) | 52 (39-87) | 1.26 (1.03-1.47) | 1.23 (1.17-1.28) | 53 (39-86) | 1.20 (0.99-1.40) | 1.18 (1.13-1.22) | 55 (39-87) |

1. Compared with normal weight (BMI 20 to <25 kg/m^2^)
2. HR_U_ refers to the effect if unmeasured confounding enlarged the hazard of CVD by a factor of 1.1 or 1.98. The hazard ratio of U on CVD was adjusted for BMI, mediators and other covariates.
3. The same values were used for unmeasured sensitivity analysis of general and central adiposity because of high correlation (r≥0.9) between the BMI ≥ 30 kg/m^2^ and WC≥90cm.
