# Supplementary material for: Contribution of obesity and cardiometabolic risk factors in developing cardiovascular disease: a population-based cohort study
Source: Sci Rep. 2022 Jan 28;12:1544. doi: 10.1038/s41598-022-05536-w (PMC8799723; doi:10.1038/s41598-022-05536-w)
Supplement: Supplementary file 4 — Supplementary Table S4. [file 41598_2022_5536_MOESM4_ESM.docx]

Supplementary Table 4

| Drug adjusted total, direct, and indirect effects of overweight and adiposity on cardiovascular diseases (CVDs) using a parametric method without considering exposure-mediator interaction. | | | | | |
| --- | --- | --- | --- | --- | --- |
| exposures | Mediators | Total effect^a,b^ | Natural direct effect | Natural indirect effect | Proportion mediated^c^  (95% CI) |
|  |  | HR (95% CI) | HR (95% CI) | HR (95% CI) |  |
| overweight | Blood pressure (mmHg) | 1.54 (1.26-1.99) | 1.43 (1.07-1.80) | 1.08 (0.99-1.18) | 20 (0-70) |
|  | Cholesterol (mmol/l) |  | 1.42 (1.13-1.90) | 1.09 (1.01-1.16) | 23 (5-54) |
|  | Glucose (mmol/l) |  | 1.44 (1.13-1.88) | 1.07 (1.01-1.16) | 18 (3-55) |
|  | Blood pressure, cholesterol, and glucose |  | 1.24 (0.90-1.58) | 1.24 (1.12-1.38) | 56 (31-100) |
| General obesity | Blood pressure (mmHg) | 1.56 (1.17-2.06) | 1.45 (1.00-1.92) | 1.07 (0.91-1.39) | 19 (0-100) |
|  | Cholesterol (mmol/l) |  | 1.17 (0.85-1.72) | 1.33 (1.16-1.63) | 69 (28-100) |
|  | Glucose (mmol/l) |  | 1.29 (0.88-1.80) | 1.21 (1.02-1.52) | 48 (5-100) |
|  | Blood pressure, cholesterol, and glucose |  | 1.08 (0.68-1.50) | 1.44 (1.14-2.03) | 85 (30-100) |
| Visceral adiposity | Blood pressure (mmHg) | 1.53 (1.23-1.93) | 1.36 (1.08-1.69) | 1.13 (1.05-1.24) | 33 (9-65) |
|  | Cholesterol (mmol/l) |  | 1.40 (1.14-1.72) | 1.09 (1.02-1.15) | 24 (3-40) |
|  | Glucose (mmol/l) |  | 1.39 (1.09-1.71) | 1.09 (1.02-1.17) | 25 (8-54) |
|  | Blood pressure, cholesterol, and glucose |  | 1.19 (0.94-1.56) | 1.28 (1.17-1.41) | 63.5 (35-100) |
|  |  |  |  |  |  |

MI: body mass index; CI: confidence interval; HR: hazard ratio; WC: waist circumference.

^a^Compared with normal-weight participants for general adiposity and WC<90 cm as a reference for central adiposity.

^b^All models were adjusted for age, gender, smoking, physical activity level, educational status, family history of CVDs, as well anti-hypertensive, diabetes mellitus, and hypercholestrolemia medications.

^c^The direct, indirect, and total effects were estimated for each bootstrap resampling
